# “Police shootings, now that seems to be the main issue” – Black pregnant women’s anticipation of police brutality towards their children

**DOI:** 10.1186/s12889-022-12557-7

**Published:** 2022-01-20

**Authors:** Renee Mehra, Amy Alspaugh, Linda S. Franck, Monica R. McLemore, Trace S. Kershaw, Jeannette R. Ickovics, Danya E. Keene, Alyasah A. Sewell

**Affiliations:** 1 Department of Chronic Disease Epidemiology, Yale School of Public Health, 60 College St, New Haven, CT 06520 USA; 2 Department of Family Health Care Nursing, School of Nursing, University of California, San Francisco, UCSF Box 0606, San Francisco, CA 94143 USA; 3 College of Nursing, University of Tennessee, 1200 Volunteer Blvd, Knoxville, TN 37916 USA; 4 Department of Social and Behavioral Sciences, Yale School of Public Health, 60 College St, New Haven, CT 06520 USA; 5 Department of Sociology, Emory University, 1555 Dickey Dr, Atlanta, GA 30322 USA

**Keywords:** Police brutality, Pregnancy, Maternal and infant health, Discrimination, Health inequities

## Abstract

**Background:**

A disproportionate number of people who are killed by police each year are Black. While much attention rightly remains on victims of police brutality, there is a sparse literature on police brutality and perinatal health outcomes. We aimed to explore how Black pregnant women perceive police brutality affects them during pregnancy and might affect their children.

**Methods:**

This qualitative study involved semi-structured interviews among 24 Black pregnant women in New Haven, Connecticut (January 2017 to August 2018). Interview questions explored neighborhood factors, safety, stressors during pregnancy, and anticipated stressors while parenting. Grounded theory informed the analysis.

**Results:**

Participants, regardless of socioeconomic status, shared experiences with police and beliefs about anticipated police brutality, as summarized in the following themes: (1) *experiences that lead to police distrust* – “If this is the way that mommy’s treated [by police]”; (2) *anticipating police brutality* – “I’m always expecting that phone call”; (3) *stress and fear during pregnancy* – “It’s a boy, [I feel] absolutely petrified”; and (4) *‘the talk’ about avoiding police brutality* – “How do you get prepared?” Even participants who reported positive experiences with police anticipated brutality towards their children.

**Conclusions:**

Interactions between Black people and police on a personal, familial, community, and societal level influenced how Black pregnant women understand the potential for police brutality towards their children. Anticipated police brutality is a source of stress during pregnancy, which may adversely influence maternal and infant health outcomes. Police brutality must be addressed in all communities to prevent harming the health of birthing people and their children.

## Background

On May 25, 2020, police murdered George Floyd, a 46-year-old unarmed, handcuffed Black man [1, 2]. Among the last words uttered by Mr. Floyd before his murder were “Momma, I love you” [3]. Although she died 2 years prior, the connection between mother and child can be transcendent. Fear of hearing these words under such circumstances was a common reality for Black mothers before Mr. Floyd’s death [4, 5], but has gained national attention in the aftermath of his murder.

In the United States (U.S.), approximately 1000 people are killed by police each year [2]; almost one-third of those killed are Black [6, 7]. While unjust killings of Black men and boys by police are highlighted in the media, unjust killings of Black women and girls also occur [8]. While attention rightly remains on victims of police brutality, police killings of Black people also affect the health of pregnant and postpartum Black people [9–13]. Yet, despite the frequency and over exposure to police brutality within Black communities [6, 7, 14], few studies have examined the impact of police brutality on perinatal health outcomes. Recent studies have found neighborhood-level fatal violence by police during pregnancy [9], and police incidents [10] were associated with an increased risk of preterm birth (< 37 completed weeks of gestation). After the police killings of Philando Castile in 2016 and Thurman Blevins in 2018, over half the women in and around Minneapolis felt these killings by police impacted their current pregnancy [11]. Among Black pregnant people, mostly with low incomes, in Atlanta, anticipated negative encounters between Black youth and police were strongly associated with prenatal depression [12]. Anticipated and vicarious negative encounters with police were associated with poorer mental health among Black women [12, 13] and Black youth [15], and negative attitudes towards police among Black girls [16]. Furthermore, 70% of Black women surveyed in Washington, D.C. (District of Columbia) reported being very concerned about their children being harmed or harassed by police [13].

Racial inequities in adverse birth outcomes are intransigent and substantial; the prevalence of low birthweight (< 2500 g) is more than two times higher among Black infants than white infants [17]. Racism plays a major role in these inequities, and are associated with increased risks of adverse birth outcomes [18–22]. For example, experiences of racism are associated with increased risks of preterm birth and low birthweight particularly among Black women [18–22]. Black women experience racism in many different contexts: throughout the lifecourse; in interpersonal (e.g., encountering gendered racial stereotypes), institutional (e.g., differential access to opportunities, goods, services, and healthy neighborhoods), and internalized (e.g., accepting racial stereotypes) forms; across different life domains, such as employment, education, health, housing, legal, and everyday social settings; both directly and vicariously, particularly through their children; and with vigilance in anticipation of future racism [23]. However, vicarious experiences of racism in relation to their children is perceived to be the most impactful experience of racism and is a major source of stress [23]. For example, Black women reported feeling stressed and hurt when their children experienced the hurt of daily racist interactions [23]. Furthermore, both direct and anticipated experiences of racism among their children were major sources of stress, anxiety and sadness, as Black women felt responsible for both protecting their children against racism and preparing them to deal with racism in the future [23]. Additionally, actual and anticipated racist experiences of Black women and their children elicits behavioral (expressing emotions or suppressing feelings), cognitive (ignoring or accepting racism), emotional (feeling stressed or worthless), and physiological (feeling sick) responses that may adversely affect the health of Black women and their infants [23].

Black women may experience multiple axes of oppression based on intersecting stigmatized and marginalized identities based on race, gender, socioeconomic status, and other factors [24, 25]. Therefore, we used an intersectional framework to explore how Black pregnant women perceive police brutality affects them and their view on how police brutality might affect their children. Anticipated racism from police, specifically police brutality towards their children, may be a distinct factor contributing to chronic stress among Black women. Understanding Black women’s experiences and perceptions of police brutality in relation to their pregnancies and children provides new insights to inform policy and clinical guidelines that may reduce racial inequities in health outcomes.

## Methods

### Study design and data collection

We conducted a qualitative study exploring the lived experience of Black pregnant women in New Haven, Connecticut [26]. Residents in Connecticut predominately identify as white (68.1%), followed by Hispanic (15.4%) and Black (9.8%) [27]. However, New Haven is a racially and ethnically diverse city with a little less than one-third of the approximately 140,000 residents identifying as Black (31.5%), Hispanic (30.4%), and white (30.3%) [27]. As with other cities and towns in Connecticut, the New Haven Police Department is less racially and ethnically diverse than the population it serves, with 25.6% of its officers identifying as Black and 20.8% identifying as Hispanic [28, 29].

Pregnant women who were at least 18 years of age and self-identified as African American or Black were eligible to participate. At the start of the study, we aimed to diversify our sample based on socioeconomic status, as experiences, including racism [30], may differ by socioeconomic status. We recruited participants via flyers posted in the community that described the aim of the study. Participants had no prior relationship with the researchers. Approval for the study was granted by the Yale University Institutional Review Boards.

We iteratively developed our interview guide, which explored neighborhood-level structural opportunities and barriers for a healthy pregnancy, neighborhood influences on health behaviors, current and anticipated sources of stress, and experiences of discrimination. Interview questions that are relevant to this analysis of women’s experience of and views on police brutality explored: *neighborhood factors* (Can you tell me what it’s like to live in your neighborhood? What do/don’t you like about your neighborhood? Is there anything that you would change in your neighborhood?), *safet*y (Can you tell me about the safety of your neighborhood?), *experiences of discrimination* (Can you recall a time when you felt that you were treated unfairly or harassed?), *current stressors during pregnancy* (Are there things that are stressful (or make you worry) during your pregnancy?), and *anticipated stressors* (As a future parent are there things that you worried about for your infant? What are you going to tell your child about growing up Black in your neighborhood?). Probing questions specifically related to experiences with police included: You mentioned the police, how are your experiences/interactions with the police? Have you had other interactions with the police and how have they been? You mentioned you’ve seen other interactions with the police, can you describe those?

The first author conducted one-time, individual, in-person, semi-structured interviews between January 2017 and August 2018 and obtained verbal consent from participants prior to the interview. Interviews were approximately 45 to 60 min in length, were conducted in a private room at the Yale School of Public Health, and were audio-recorded and transcribed by the first author and a transcription company. The first author verified transcriptions against audio recordings to ensure the accuracy and completeness of transcripts. We did not return transcripts to participants for comment or corrections. Participants received $40 for their time and contribution.

### Analysis

We used traditional grounded theory strategies to analyze and interpret data. Grounded theory is a constellation of methods used to iteratively and inductively move back and forth between data, rigorously compare data, and develop theoretical analyses to inform policy and practice [31]. Three researchers open-coded selected small samples of interviews in order to develop concepts and codes. Codes were derived from the data and interview guide, and grouped into domains to establish a codebook. Data saturation was achieved as no new codes emerged. Researchers used double coding to establish consistency of coding and to iteratively refine the codebook. Coding issues were resolved through consensus.

Transcripts of interviews were imported into ATLAS.ti software (Version 8, Scientific Software Development GmhH, Germany, 2018) for electronic coding. The first author applied the final codebook to all transcripts. The research team met to discuss findings, monitor biases, and engage transparently about the data. The first author wrote field notes after conducting interviews and memos after research meetings about developing themes. For this analysis, we used codes in the domain of police. Participants did not provide feedback on findings.

The authors are trained and experienced in qualitative research methods and identify as being white, Black, and biracial (Asian and white). At the time of the study, we were doctoral students, postdoctoral scholars, midwives, nurses, and professors in nursing, public health, and sociology. We acknowledge not all people with the capacity for pregnancy identify as women. Participants referred to themselves as women and mothers; therefore, we use these terms in reporting our research findings.

## Results

There were 24 participants (aged 21 to 45 years, median 32 years), none of whom refused to participate or dropped out of the study. Over half of the participants (*n* = 14; 58%) indicated pregnancy for the first time. Ten participants (42%) received public assistance benefits as their primary source of income and fourteen participants (58%) were supported by employment-related income generated by themselves, their partner, or families. In response to questions about structural barriers, stress and discrimination, 19 participants (79%) spontaneously mentioned the police without being directly solicited. Participants described interactions between Black people and police on a personal, familial, community, and societal level, and feelings and thoughts that shaped their beliefs regarding whether they and their children would be protected by police or whether they or their children would experience police brutality in the future. From these narratives, we identified four overarching themes: (1) *experiences that lead to police distrust* – “If this is the way that mommy’s treated [by police]”; (2) *anticipating police brutality* – “I’m always expecting that phone call”; (3) *stress and fear during pregnancy* – “It’s a boy, [I feel] absolutely petrified”; and (4) *‘the talk’ about avoiding police brutality* – “How do you get prepared?”

### Experiences that lead to police distrust – “If this is the way that mommy’s treated [by police]”

Participants, regardless of socioeconomic status, shared how both personal experiences and stories of friends and family shaped their relationship with police. Experiences included stories from their youth, stories about police interactions when living in predominantly white spaces, and stories, often from male family members, of mistreatment, racial profiling, and overt racism by police. Though no interaction singularly formed a narrative, personal experiences were placed within a larger historical and social context of police brutality within Black communities.

Participants reported positive and negative experiences with police that shaped their relationship with law enforcement. A few participants described only friendly and respectful interactions with police. However, many participants discussed having both positive and negative interactions with police. For example, participants discussed interactions with police that made them feel safer because of quick police response or being comforted by the presence of a police station near their house, yet they also described difficult interactions with police which negatively influenced their trust of police. One participant recalled a story from several years ago when police, thinking she fit the description of a suspect they were looking for, harassed her and her friends. She did not think this interaction was due to racism and described her current relationship with police as friendly. But when asked about her comfort level calling police for help, she replied: “Whatever needs, we need help with or something like that, we try to take care of it on our own before calling police, unless it’s an emergency.” (21-year-old, first-time pregnant woman). Although the original incident with police was viewed in hindsight as benign, it influenced her ability to trust police.

Another participant discussed how recent interactions with police had created a lack of trust. Upon moving to an overwhelming white neighborhood, one participant revealed that police had gone through her trash for the first year and a half that she lived there. She reported feeling accepted by police at this point, but then went on to talk about yet another negative interaction with police, which she described as being racist. When she called police to her house to deal with her alcoholic husband, they did not deal with her drunk husband who was white. Instead, they inquired as to whether her name was on the house and then asked her to leave the house with her infant and while pregnant.

Other participants who had experienced positive interactions with police shared stories of mistreatment experienced by predominantly male friends and family at the hands of police. Stories included men who were trailed by police for their mere presence in white neighborhoods, and, “Getting pulled over, being asked, you know, show your I.D.; ‘Why are you here?’ type of questions.” (34-year-old, pregnant mother of one). Another participant shared a story about a friend in New York who had been murdered by police at his home during an exacerbated episode of mental illness. Taken as a whole, personal experiences and shared stories shaped participants’ perception and trust regarding police.

### Anticipating police brutality – “I’m always expecting that phone call”

Personal and shared stories of both positive and negative experiences with police, provided a basis from which participants conceptualized interactions with police. This conceptualization contributed to participants anticipating future interactions, both for themselves and notably, for their children. Even for participants who reported positive personal interactions with police, fear of police brutality toward their children was present.

One participant shared she had always been nervous about the potential for her three younger brothers to fall victim to police brutality, saying: “But in the back of my mind it’s like I’m always expecting that phone call.” (31-year-old, first-time pregnant woman). Now pregnant with her first child, she sees this anticipation transferring to her child. She expressed concern about how pervasive and random violence seems to be currently, especially from police, saying: “It seems like you can’t really go anywhere without something happening.” This concern regarding police brutality in the community was vocalized by many participants, even among those who reported little problem with police brutality or safety in their own lives, and those who had previous positive interactions with police.

The participant who shared stories about police looking through her trash and mistreating her during a domestic disturbance call was attune to how this reality would unfold for her children. In anticipation of the potential for police brutality, she was planning to move her and her children, who are biracial, to another neighborhood, saying: “If my son who’s ever in trouble for any reason or just in the wrong place at the wrong time, I don’t feel that he has that protection in our neighborhood.” (36-year-old, pregnant mother of one). For many participants, realities of life as a Black person, and especially considering the future for their Black son, necessitated anticipatory planning during pregnancy.

### Stress and fear during pregnancy– **“It’s a boy, [I feel] absolutely petrified”**

Several participants described the anticipation they felt regarding police brutality against their children as a source of stress during pregnancy. Some participants who knew they were having a son, reported having a heightened sense of fear about raising their son and how their son would be treated by police. Again, both participants who had and had not experienced discrimination or profiling by police shared a common concern about how police might treat their male children and already felt the weight of this anticipatory police brutality during their pregnancies.

One participant described several friendly interactions with police throughout her life. But when asked about her biggest concerns raising a child, she questioned: “What would their experience be with the police?” (28-year-old, first-time pregnant woman). Discrimination and the possibility for brutality from police, were constantly on her mind during pregnancy.

Another participant discussed how she considered the potential for ethnoracial marginalization to impact her son’s life, where his everyday experiences could be typified to signal a racial character to his everyday practices and experiences that categorizes, stratifies, and marginalizes him [32]. “A lot of [Black men] are being killed by policemen. A lot of them are being targeted, you know just because of the color of their skin and you know a lot of them are going to jail because of things they may have not even done you know. So, I don’t know, I just, I guess, I’ll just protect him as much as I can.” (21-year-old, first-time pregnant woman). This participant, like many others, felt the disconnect between her ability to prepare and protect her child at home and the reality of racism and police brutality in the larger world. Limits of parental protection in a racist world were a source of stress, fear, and anxiety during pregnancy. Similarly, another participant said: “How do you protect them or how do you be OK sending them out in the world where they should be safe. And that may or may not be the reality.” (31-year-old, first-time pregnant woman).

For others, it was the seeming randomness of police brutality against Black people that was stressful. One participant was concerned not about her community specifically, but about the fear and stress of raising a biracial son, who is going to have darker skin, in society at large. When reflecting on national news, she said: “That kind of makes you stop and think of, you know, how do you prepare your child to go out in the world at this point in time, when they may not be doing anything wrong? They might just be the wrong place at the wrong time or make a wrong move. And you know there’s a shooting or whatever the case may be. So, things like that I think you know definitely make you a little bit scared.” (34-year-old, pregnant mother of one). Again, the gap between a parent’s protection and the possibility of police brutality was a source of stress during pregnancy.

### *‘*The talk’ about avoiding police brutality – “How do you get prepared?”

Participants had already begun thinking about and planning for having ‘the talk’ about police brutality and racism with their children, even when their children were in utero. The necessity of having this conversation with their children was almost universal. Many struggled with worries about when to have it, how much information to divulge, and who to involve.

One participant, who already had an 8-year-old son, was wrestling with these decisions in a much more urgent manner. Although she did not feel there was an issue with violence in her neighborhood specifically, she expressed a concerned about the community at large and the need to prepare her child: “It’s a discussion that needs to be had. I personally don’t know what I’m gonna say yet, I don’t even know when I should have that conversation because he’s so young or the baby’s so … So, yeah, I don’t know, that’s tough.” (31-year-old, pregnant mother of one).

When asked about what she worries about as a Black mother of a Black child, one participant said her child’s safety was something she was already very worried about. ‘The talk’ was one vehicle to provide some source of protection. As a first-time pregnant participant said: “I think about especially lately in the news with police brutality as well, that’s something that I have to think about when that time comes to have that conversation with him. Yeah, I do think about that, his safety.” (25-year-old, first-time pregnant woman).

Another participant struggled with how to approach the topic. She herself discussed not having received ‘the talk’ growing up because it wasn’t a conversation that needed to be had in her community. To her, it was critical to strike the right note: “I want to have the right balance. Like I don’t know what it is, but I don’t want to freak my child out completely. But it’s like they need to know right.” (31-year-old, first-time pregnant woman). Figuring out how to balance the weight of talking to children about racism and police brutality is an immense burden for anyone, especially during pregnancy.

## Discussion

We found for participants in our study, anticipation, fear, and stress regarding police brutality toward children occurred before their children were even born. Perceptions of how police may treat their children were based on personal, familial, community, and societal level influences. Perceptions of and experiences with police did not differ by socioeconomic status. Even participants who reported positive experiences with police anticipated brutality towards their children.

Many of our findings are supported by and expand upon existing literature. Participants in this study discussed an overwhelming feeling of stress and concern related to safety of their future children at the hands of police. One study found Black mothers experienced pervasive vigilance by consciously preparing themselves for racist encounters on a daily basis [23]. In our study, preparation and vigilance during pregnancy manifested as concerns about violence by police against children. Another study found that among Black pregnant people, anticipation of negative police interactions was significantly associated with prenatal depression [12]. Participants in our study expressed feelings more aligned with stress and anxiety than depression, showing that responses to anticipated violence vary and often exist simultaneously.

In our study, attitudes of Black pregnant women towards police were not uniformly positive or negative, but instead were often complicated and nuanced. Many participants reported positive personal interactions with police, saw the presence of a police station in their neighborhood as a positive attribute, and discussed certain types of police (school and campus police, in particular) with great fondness. However, some of these same participants also vocalized a fear of police mistreatment related to their community and their current and future children. Complex feelings toward police and police protection indicated the importance of the interplay between personal lived experience and experiences of others, either family members or close friends, or on national news. This seemingly contradictory view of police as both providing protection and causing harm is noted in larger, national polls [33]. These attitudes may indicate that police reform, instead of abolition, may be a preferred route by participants in our study. It may also indicate a population attempting to protect themselves with the lesser of two bad options: police and neighborhood violence.

### Strengths and limitations

The strengths of this study include exploring perceptions about and experiences with police from a sizable sample of both pregnant and parenting Black women, some of whom know the sex of their fetus. One component of neighborhood context as it relates to pregnancy that we explored was safety; however, it was not the focus of the study. Despite this, the majority of participants (79%) initiated discussion on the topic of police. However, more targeted questions on policing may have generated different results. Furthermore, racial discordance between interviewer and participants may have influenced experiences participants shared. Participants’ experiences may not be transferable to other settings with different racial and ethnic composition of the population and police workforce.

### Public health implications

Participants reported anticipating police brutality against their unborn children and how to best prepare their children to avoid police brutality caused stress and fear during pregnancy. Stress specifically related to constant police surveillance and fear of violence from police creates “surveillance stress” [34]. This stress may affect people with the capacity for pregnancy in unique ways. Using neighborhood-level exposure to lethal policing in New York, Sewell and colleagues identified an increase in risk of high blood pressure and obesity among people living in lethally surveilled areas [14]. Associations were not experienced equally by gender, however, with women having a 30 to 54% greater risk of high blood pressure, obesity, and diabetes compared to men [14]. Associations between police contact and police brutality and adverse birth outcomes may differ by ethnoracial marginalization, with Black birthing parents having a greater risk of preterm birth than non-Black birthing parents [9, 10].

To provide the best care for pregnant people who may be experiencing stress related to police brutality, clinicians should thoughtfully screen all pregnant people using validated screening tools [35]. Pregnant people who are identified as experiencing high levels of stress should be provided support and linkage to individuals with experiences treating mental health related to racial trauma and/or other culturally informed therapies of postpartum depression, assessed for adequate social support, and provided positive coping strategies. However, these interventions are insufficient to address the root cause of this stress. Participants in our study were aware of the limits of their own ability to keep their future children safe; as individuals they were unable to mitigate the effects of a problematic law enforcement system. Systemic problems necessitate systemic solutions – police brutality, not people’s concern about this brutality, is the problem that must be solved.

### Policy implications

A reimagination of policing may help to address police brutality. Legislatively, the George Floyd Justice in Policing Act of 2020 would increase accountability for police misconduct, restrict the use of specific policing practices, improve transparency and data collection, and implement best practices and training requirements [36]. The bill passed the House of Representatives on June 25, 2020 and has failed to advance in the Senate [36].

Multisector collaborations, such as police–public health collaborations, are another avenue to improve policing. One study found police officers with crisis intervention training were less likely to arrest a person and were more likely to make referrals for mental health services or transport the person to a treatment facility than police officers without this training; however, there was no difference in use of force by crisis intervention training status [37]. Training programs and community activities are suggested as a solution to minimize implicit bias among police officers [38]. However, building strong community-police relationships will not be sufficient given that participants in this study who reported positive experiences with police still anticipated brutality towards their children. Participant’s positive experiences with police did not outweigh their own negative experiences with police nor the experiences of police brutality among friends, neighbors, and community members locally and nationally. As a national public health crisis, police brutality must be addressed in all communities using community-centered strategies [39] to prevent harming the health of birthing people and their children.

## Conclusions

This study illuminates that the fear of police brutality starts before a child is even born, potentially influencing the health of the birthing person and child through a host of mechanisms that can create deleterious effects lasting decades. Police brutality is but one symptom of systemic racism within the carceral system. Multilevel interventions will be required to address police brutality and its potential adverse effects on maternal and infant health.

## Data Availability

The datasets used and/or analyzed during the current study are not publicly available due to the sensitive nature of the interviews, but are available from the corresponding author on reasonable request.

## Abbreviations

I.D: Identification
D.C: District of Columbia
U.S: United States
